# Improving the first-line treatment of febrile illnesses in Ghana: willingness to pay for malaria rapid diagnostic tests at licensed chemical shops in the Kintampo area

**DOI:** 10.1186/s12962-018-0090-2

**Published:** 2018-01-31

**Authors:** Theresa Tawiah, Keziah Malam, Anthony Kwarteng, Constance Bart-Plange, Lawrence Febir, Vivian Aubyn, Konrad Obermann, Seth Owusu-Agyei, Kwaku Poku Asante

**Affiliations:** 1 Kintampo Health Research Center, P. O Box 200, Kintampo, Ghana; 2 National Malaria Control Programme, Ghana Health Service, Accra, Ghana; 3 Mannheimer Institut of Public Health, Heidelberg University, Heidelberg, Germany

**Keywords:** Malaria, Malaria RDT, Willingness to pay, Willingness to sell, Kintampo, Ghana

## Abstract

**Background:**

Use of malaria rapid diagnostic test (mRDT) enhances patient management and reduces costs associated with the inappropriate use of antimalarials. Despite its proven clinical effectiveness, mRDT is not readily available at licensed chemical shops in Ghana. Therefore, in order to improve the use of mRDT, there is the need to understand the willingness to pay for and sell mRDT. This study assessed patients’ willingness to pay and licensed chemical operators’ (LCS) willingness to sell mRDTs.

**Methods:**

The study was a cross-sectional survey conducted in Kintampo North Municipality and Kintampo South District of Ghana. Contingent valuation method using the dichotomous approach was applied to explore patient’s willingness to pay. In-depth interviews (IDIs) were used to obtain information from licensed chemical operators’ willingness to sell.

**Results:**

Majority 161 (97%) of the customers were willing to pay for mRDT while 100% of licensed chemical operators were also willing to sell mRDT. The average lowest amount respondents were willing to pay was Ghana cedis (GH¢) 1.1 (US$ 0.26) and an average highest amount of GH¢ 2.1 (US$ 0.49). LCS operators were willing to sell the test kit at an average lowest price of GH¢1 (US$ 0.23) and average highest price of GH¢2 (US$ 0.47).

**Conclusion:**

Community members were willing to pay for mRDT and LCS operators are willing to sell mRDTs. However, the high cost of the mRDT is likely to prevent the widespread use of mRDT. There is a clear need to find system-compatible ways to subsidize the use of mRDT via National Health Insurance scheme.

## Background

Malaria kills almost one million people every year and affects about half a billion people globally. In 2014 malaria led to about 214 million cases worldwide and about 395,000 deaths in Africa alone [1]. Malaria is one of the leading illnesses in Ghana and the main cause of mortality and morbidity, accounting for 44% of all outpatient visits, 37% of all admissions and 11% of deaths in the public health facilities [2]. Malaria continues to pose economic burden on the health systems although the incidence of malaria has decreased. In sub-Saharan Africa, an average of US$ 300 million is spent on malaria case management each year out of which 23% is spent on commodities for diagnosis and treatment [3]. The distribution of antimalarials and use of diagnostic tests at the community level by community medicine distributors is one of the interventions which has been adopted to reduce mortality from malaria [4].

Until recently, the diagnosis and treatment of malaria had been presumptive due to lack of diagnostic tools in many health facilities [5, 6]. The practice of presumptive malaria diagnosis in Africa has led to over-diagnosis of malaria [7–11], over-prescription of antimalarials, and undiagnosised or inappropriately treated non-malarial febrile illnesses (NMFI) [9, 11]. As a result, the World Health Organisation has revised the fever case management guidelines and now recommends confirmed malaria diagnosis before artemisinin-based combination therapy (ACT) [12].

In most countries in Africa, large proportions of the population utilize the private sector, and in particular licensed chemical shops as their first option for treatment of fever and malaria [9, 13, 14]. Similar treatment-seeking behaviour is seen in Ghana, where an estimated 50% of patients, visit drug shops as their first point of care [14–17] where malaria is diagnosed presumptively.

Malaria rapid diagnostic test (mRDT) has considerable potential to improve the diagnosis of malaria [18]. Its use in malaria diagnosis is increasing in many countries as a result of their ease of use with minimal training [19]. Using mRDTs for diagnosis at the community level will shorten the time between onset of symptoms and initiation of appropriate treatment. Use of mRDT will help reduce costs by avoiding unnecessary prescription of expensive antimalarials and ultimately prevent drug resistance among patients, thus reducing other potential future costs.

Despite its importance, mRDTs have not yet been included in the list of commodities that registered drug shops can provide as part of their service under the national health insurance scheme in Ghana. Therefore, LCS operators will have to sell mRDTs to community members for malaria management.

In order to improve the use of mRDTs, there is a need to understand issues related to willingness to pay for and sell mRDTs. This study assessed the willingness of patients to pay for and LCS operators to sell mRDTs.

## Methods

### Study design

The study was a cross-sectional survey that employed both quantitative and qualitative techniques. Data were collected between February 2013 and December 2013 among customers and LCS operators in the Kintampo area of Ghana.

### Study area

The study was part of a survey which accessed the feasibility of test-based management of malaria by LCS operators using mRDT [20]. In the study, participating LCS were provided with mRDTs at no cost and sold it to the patients at subsidized price. The study was conducted in Kintampo North Municipality and Kintampo South District in the middle belt of Ghana. The districts covers an area of 7162 km^2^ with resident population of approximately 134,970 [21]. There are two main seasons: the rainy season from March to November and dry season from December to February. The population are subsistence farmers involved in the cultivation of yam and maize with increasing activity of petty trading. There are two [2] hospitals, 12 health centres/clinics and 26 Community Health–Based Planning Services (CHPS) compounds. There are three [3] pharmacies and about 68 LCS in the studied area.

### Quantitative data collection methods

The survey was conducted as exit interviews among LCS customers seeking treatment for fever or malaria. Participants were identified as they exited the LCS after seeking treatment and their address recorded if they we willing to be part of the study. They were later followed–up within 48 h in their homes for the interviews to be conducted to ensure confidentiality and provide a convenient place for the interview without any influence from the LCS operators. Each LCS operator was visited once by the study team and the first 4 clients to the LCS who were willing to be part of the study were included in the exit interviews.

Contingent valuation method (CVM) was used to assess customers willingness to pay using dichotomous approach, which is either yes to willingness to pay or the opposite [22]. This approach was appropriate for this study because at the time of the study, LCS operators did not sell mRDTs in the study area. Monetary values were determined by asking how much respondents were willing-to-pay at a highest and lowest price to have the commodity.

The CVM questionnaire was categorized into three main groups. Firstly, respondents were asked questions on their demographics characteristics such as age, sex, educational level, marital status and religious background. Secondly, the questions elicited respondents’ willingness to pay for mRDTs that was used to diagnose malaria during their visit to the LCS. The highest and lowest prices respondents were willing to pay was assessed with the assumption that LCS operators would sell mRDT. Thirdly, respondents’ socio-economic status were assessed using household durable and non-durable assets such as ownership of land and farm, motorcycle, car, bicycle, radio, household building material for well and floor, roofing material, electricity at homes, etc. These household assets were used to generate household asset scores. Eigen-values were computed using principal component analysis, the assets were weighted and grouped into various components. The first component was used to generate the wealth tertile for participant’s household. This procedure is similar to what was done in previous studies [23–25].

### Qualitative data collection method

Focus Group Discussions (FGDs) were conducted among community members in the community where the selected LCSs were located to assess the communities’ perception of cost of treatment at LCS. An interview guide was used to assess reasons for choosing to pay for a certain amount and factors influencing their choice. Emerging themes relevant to the objectives of the study were explored. In-depth interviews (IDIs) were conducted among LCS operators to determine how much they are willing to sell mRDT. IDIs were also conducted among managers of National Health Insurance Scheme (NHIS) to assess potential ways of incorporating mRDTs into the scheme.

### Sample size

Forty-two out of the 68 LCS in the study area were in good standing with the Pharmacy Council (the national regulatory body of LCS) and were included in the study. A minimum of 4 respondents was interviewed from each LCS for logistical reasons. A total of 169 respondents were therefore included in the WTP estimation. This number provides over 90% power (at α = 0.05) based on an assumption of a WTP estimation of about 80% from a previously report WTP estimate of 90% [26]. Seven FGDs (four with females and three with males), three IDIs among LCS operators and six IDIs with NHIS staff were conducted.

### Data collection, management and analysis

A structured questionnaire was used for data collection. Data collection tools were pre-tested prior to its administration. All completed data collection tools were received in the Kintampo Health Research Centre computer laboratory. Data entry was carried out using Microsoft Excel software and transferred to Stata software version 13 (Stata Corp, Texas, USA). Qualitative data from FGDs and IDIs were recorded using digital tapes, transcribed and managed with the QRS NVIVO software version 8. Categorical demographic data such as educational status, sex and socioeconomic were summarised as percentages, while continuous variables such as age was summarised as means together with their standard deviations.

The outcome measure was defined as respondents’ willingness to pay for mRDT but not dependent on a set cost of the mRDT. A secondary outcome measure for univariate and multivariate analysis was defined as respondent’s willingness to pay for mRDT at a defined cost of ≤ GHC 2.00; the price at which LCS sold one mRDT. A logistic regression to explore the factors associated with willingness to pay for mRDT was also performed. The dependent variable for the regression analysis was WTP status which was expressed as yes or no. Variables that were found to be associated with WTP in relevant studies or variables that were significant at p < 0.25 in the univariate analysis were included in the multiple logistic model as independent variables (Table 1).

**Table 1:** Definition of variables

Variable	Definition
WTP status	Dummy variable = 1 for yes, 0 for no
Age	Age of respondents in years 16–25 = 1, 26 and above = 2, don’t know = 4
Educational level	Level of education of respondent Non-formal = 1, formal = 2
NHIS status	Insurance status dummy variable 1 = insured, 0 = uninsured
Socio economic status	Highest = 1, middle = 2, lowest = 3
Marital status	Single, divorced = 1, Married/living together = 2
Gender	Male = 1, female = 2
Number of children	Under 5 = 1, 5–15 = 2, no child = 3

## Results

### Demographic characteristics

A total of 169 clients attending LCS were recruited to participate in the study. Four (2.4%) clients refused participation into the study. Seventy-four (44.9%) respondents were males (Table 2). 60.6% of the respondents were married. The mean age of the respondents was 30.8 years [standard deviation (SD), 15.4 years]. 53.9% of participant lived in households which had < 5 children. 42.0% (70) of the respondents had not had any formal education. Fifty-four percent of the respondents had a valid health insurance i.e. were able to access health care with their health insurance under the National Health Insurance Scheme.

**Table 2:** Demographic characteristics of respondents (N = 165)

Variable	Number of respondents	Percentage
Gender		
Male	74	44.9
Female	91	55.1
Marital status		
Single/divorced	49	29.7
Married/living together	116	70.3
Age group		
16–25	50	30.3
26 and above	102	61.8
Don’t know	13	7.9
Number of children in household		
Under 5	89	53.9
5–15	41	24.9
No child	35	21.2
Socio-economic status		
Highest	55	33.3
Middle	55	33.3
Lowest	55	33.3
Form of education		
Non-formal	70	42.4
Formal	95	57.6
Religious background		
Christian	90	54.6
Muslim	68	41.2
Other	7	4.3
Insurance status		
Insured	89	53.9
Non-insured	76	46.1

### Willingness-to-pay

96.4% (160) of the respondents affirmed their willingness to pay for mRDT. Three of the respondents who were not willing to pay for mRDT stated that they had valid health insurance and therefore, insurance should cover the cost of mRDT at LCS just as is being done at health facilities. The other two said the cost of mRDT was too high. The mean highest and lowest selling price offered by respondents to pay for mRDTs was GH¢ 2.10 (US$ 1.05) (SD GH¢ 1.90, range 0.20–15.00) and GH¢ 1.10 (US$0.55) (SD GH¢ 0.99, range 0.10–5.00) respectively. Exchange rate as at October 2013: US$ 1 is equivalent to GH¢ 2 [27]. Only 3 (1.9%) respondents were willing to pay above GH¢ 7.00 (US$3.5).

### Factors associated with willingness to pay at the selling price of mRDT

Table 3 describes respondents who were willing to pay for mRDT at a selling price of ≤ GHC 2 and those who were willing to pay above this selling price. 61.6% of respondents who were willing to pay for mRDT at the selling price of ≤ GHC 2 were aged 26 and above (Table 3). There was no significant differences in the characteristics of respondents who were willing to pay for mRDT at a selling price of ≤ GHC 2 compared with that of participants who were willing to pay > GHC 2.

**Table 3:** Frequency distribution and analysis of WTP at different price level

Respondent characteristics	WTP		p value
	WTP ≤ GHc2.00 N = 125 n (%)	WTP > 2.00 N = 35 n (%)	
Gender			
Male	53 (42.4)	18 (51.4)	0.34
Female	72 (57.6)	17 (48.6)	
Age group			
16–25	38 (30.4)	10 (28.6)	0.98
26 and above	77 (61.6)	22 (62.9)	
Don’t know	10 (8.0)	3 (8.6)	
Level of education			
Non-formal	52 (41.6)	15 (42.9)	0.89
Formal	73 (58.4)	20 (57.1)	
Marital status			
Single/divorced	37 (29.6)	10 (28.6)	0.91
Married/living together	88 (70.4)	25 (71.4)	
Number of children			
Under 5	70 (56.0)	17 (48.6)	0.69
5–15	30 (24.0)	9 (25.7)	
No child	25 (20.0)	9 (25.7)	
Socio-economic status			
Highest	42 (33.6)	10 (28.6)	0.83
Middle	41 (32.8)	13 (37.1)	
Lowest	42 (33.6)	12 (34.3)	
Insurance status			
Insured	67 (53.6)	18 (51.4)	0.82
Non-insured	58 (46.4)	17 (48.6)	

### Factors associated with WTP for mRDT

None of the respondents basic demographic characteristic were found to have an association with respondent’s willingness to pay for mRDT. Gender, educational level, marital status, number of children, insurance status, socio-economic status and age group were however included in the multiple logistic regression (Table 4) since they were found to have an association with the outcome from other studies. Females, respondents with higher age group, having a formal educational background and married were associated with a higher odds of willing to pay compared to males, respondents with lower age group, having no educational background and single when insurance status, socio-economic status and the number of children in a household are adjusted for. This was however not statistically significant.

**Table 4:** Multiple Logistic regression showing the relationship between respondent’s willingness to pay and their demographic characteristics

	Odds ratio	S. E	p value	95% CI
Gender				
Male	1			
Female	5.20	6.15	0.16	0.51–52.85
Age				
Below 26 years	1			
26 years and above	2.68	3.54	0.46	0.20–35.74
Educational level				
No formal education	1			
Formal education	2.01	2.67	0.60	0.15–27.39
NHIS status				
No	1			
Yes	0.17	0.21	0.15	0.14–1.92
Socio-economic status				
Lowest	1			
Middle	0.94	1.42	0.96	0.49–18.19
Highest	0.16	0.22	0.18	0.11–2.36
Marital status				
Single	1			
Married	3.47	4.81	0.37	0.23–52.35
Number of children				
None	1			
1–5 children	0.34	0.74	0.62	0.01–24.15
6 children and above	0.18	0.44	0.49	0.01–23.17

*S. E* Standard error, *CI* confidence interval

#### Reasons why respondents were willing to pay for mRDT

Most of the respondents were happy about LCS operators conducting the mRDT and were willing to pay for it. Respondents confirmed that they were previously treated presumptively because mRDT were unavailable. They appreciate that mRDTs will help improve the quality of treatment at LCS.

*“It’s good because people think once their head aches then they are suffering from malaria which might not be true so if the test is been done it will let the drug store owner or the one administering the drug knows whether the patient is suffering from malaria or not and treat the person as such”. [FGD male respondent 6, Apesika]*

Most respondents agreed to pay for mRDTs for malaria management at the LCS because they will save lot of time from queuing at health facilities and they also expect to get well as early as possible to attend to their farm activities.

*“It is good because if you are healthy you can do anything so I think if we pay for the test and we are treated on time, we will get the energy to continue with our work. It will also save us time from going to health facilities.” [FGD female respondent 3, Amoma]*

#### Reasons why respondents were not willing to pay for mRDT

Respondents who were not willing to pay for mRDT at LCS were mainly concerned about poor quality of malaria management in LCS if LCS attendants are untrained. Respondents who had subscribed to NHIS preferred to assess malaria management at NHIS accredited health providers to benefit from their subscription. Few of the respondents also stated that it will be difficult to pay for the test kit because of financial constraint.

*“…………………Drug shops are not operated by one person and therefore the person who received the training will not be the same person to test us with the kit” [FGD female respondent 1, Anyima]*

*“I will rather use my insurance because my insurance has not expired” [FGD male respondent 4, Apesika]*

*“You know for the people living in the smaller communities most of us are farmers and we only get money during the harvesting period so I think if the test is free it will be good so that we will buy the drugs as we used to.” [FGD male respondent 7, Gruma]*

### Willingness to sell

LCS operators were generally happy and willing to sell mRDT to their customers because it will help them sell the right medication to their customers. They suggested a lowest selling price of GH¢ 0.50 and highest price of GH¢ 2.00.

*“the lowest cost should be GH¢ 0.50 and the highest price that the RDT must be sold is GH¢ 1.00. This will help the patients to afford the cost in addition to the drugs.” [IDI LCS operator 1]*

However, there were some challenges that were identified that may affect mRDT sales at the LCS. They perceived irregular supply and stock outs of mRDT to be a major barrier to effective management of malaria. This potential barrier may also make customers lose confidence in LCS operators.

*“The difficulty in restocking is the problem of distance and the frequency of supply. Assuming I am supplied one box of malaria drugs today and it get finish today I still have to wait for the next supply period to be able to restock and that is the challenge. We don’t actually get supply daily and frequently because some suppliers come even after one month of supply so we always have to wait between these periods”. [IDI LCS operator 3]*

LCS operators were concerned about the affordability of mRDTs by their customers who are mainly rural and of low socioeconomic level. They indicated the cost of malaria management will be higher if the cost of mRDTs is added to the already high cost of ACTs.

*“I suggest that we sell the test kit at one Ghana cedi so that people can get enough to buy the accompanying drugs after the test”. [IDI LCS operator 2].*

### mRDT under NHIS

The NHIS staff found it important to register mRDT under the NHIS as one of the services that LCS can provide since LCS are the first point of contact for health care in the community. However, they perceive some challenges when LCS operators are allowed to sell mRDT. NHIA deals with government institutions and private registered companies but in general, some of the LCS are not registered or are not in good standing with the Pharmacy Council of Ghana.

*“the LCS operators should be registered with the Pharmacy Council and then they can be accredited under the NHIS so that incase of any legalities and malfeasants, they can be taken to court” (NHIS staff respondent 1)*

Another challenge that respondents spoke about was that NHIS is migrating from paper based to an electronic system of operations including claims and reimbursement. The NHIS staff perceive the system to be complex for the education level of LCS operators and also resource intensive as outlined below.

*“……..those who will be permitted to sell the mRDT under NHIS should be LCS operators who are properly trained and is capable of handling the biometric issues”. [NHIS staff respondent 2].*

NHIS intends assign each subscriber to specific NHIS accredited health service providers. NHIS subscribers have a choice in selecting a service provider. Therefore, it is unlikely that NHIS subscriber will choose LCS as their preferred choice for health care over other higher level health facilities. The NHIS staff perceive the preference of larger hospitals to be a challenge for LCS if they are to be accredited by NHIS as service providers for malaria management. The

*“under the capitation it will be very difficult for people to choose drug shops as their primary provider because if you seek care from them and they are not able to help where will you go?” [NHIS staff respondent 1]*

## Discussion

This study assessed community members’ willingness to pay for malaria rapid diagnostic test (mRDT). The majority of respondents (97%) were willing to pay for mRDTs at the LCS but at a price lower than its actual selling price (< GH¢ 2). To ensure test based malaria management at the community level, the price of mRDT should therefore be less than the cost price of mRDTs [GH¢ 7.3 (U$ 3.7)] at the time of the study. This finding is consistent with a related study carried out in Uganda where a majority of respondents were unwilling to pay a price higher than the subsidized price of US$ 2.49 [28]. This observation is however different from a similar study conducted in Nigeria [26], where the authors found respondents willing to pay higher price than the selling price. The difference may be because Nigeria’s per capita income ($ 2966) is higher than that of Ghana ($ 1846). The lower price placed on mRDT by the respondents was mainly due to their inability to afford mRDT and ACT at the same time. Another factor was that malaria test is done at no cost in health facilities as part of the benefit of the NHIS and therefore, mRDT should also be free at LCS. These factors were different from findings of Rennie et al. [29] where a low price was offered for mRDT because malaria was not perceived a serious disease compared to other illness such as syphilis and HIV. If community members are encouraged to access mRDT at affordable prices in the drug shops, it will help reduce over utilization of anti-malarials and also lead to appropriate treatment of other febrile illnesses. Hansen et al. concluded in their study that the subsidized price of mRDT should be lower than the subsidized price of ACT to ensure a large percentage of community members to patronize mRDT at LCS [28].

To enable majority of community members patronize mRDT at LCS, the government and other donor agencies should subsidize the mRDT as it has been done for other interventions like insecticide treated nets, childhood immunizations. Recently ACTs were subsidized by special programmes such as Affordable Medicines Facility for Malaria (AMFm) to enhance accessibility and affordability of ACTs at the community level. This program has led to a sustained reduction of the cost of ACTs in Ghana and other African countries [30–32]. These programmes were resistant to escalating cost of ACTs that arise of exchange rates. A similar program for mRDTs will enhance the quality of malaria management at the community.

The respondents in our study recognized the benefits malaria diagnosis with mRDT at the LCS and found the approach acceptable. Their perception of a benefit was expressed mainly in terms of economic gains such as time saving for their economic activities. This observation was similar to other studies [26, 28]. This perceived benefit is an important observation to use as a communication message on the importance of mRDT.

In determining factors associated with WTP using the logistic regression, respondent’s household with larger number of children were associated with a lower odds of willing to pay which can be interpreted as a lesser willingness to pay for mRDT as the number of household children increases. This is contrary to the expectation that the more children one has, the more willing one will be to pay for mRDT. Though not statistically significant, demographic variables such as females, increasing age, better education and being married had a higher odds with respondents’ willingness to pay for mRDT. This finding is similar to that found in other studies in Uganda, and Tanzania [28, 29]. In these studies, demographic characteristics such as educated respondents were more likely to pay for mRDT than uneducated respondents. Health promotion interventions should therefore target demographic groups who are less likely to pay for mRDT to ensure appropriate management of mRDTs.

All LCS operators were willing to sell mRDT and use it as part of their malaria management. It is likely they are motivated both by the potential improvement in the quality of malaria diagnosis when using mRDT as well as by having a (higher) profit through markups on the mRDT. There is therefore the need to maintain or enhance their motivation to use mRDTs by (i) ensuring an adequate mRDT supply chain to the LCS and (ii) supporting a selling price that at least does not leave the LCS operators worse off.

NHIS staff found it acceptable to integrate the mRDT as part of malaria management at LCS, but pointed out challenges in terms of health insurance data management at the LCS and the new NHIS reimbursement model. NHIS service providers are required to provide data using a data management tool that requires internet and computers. This challenge could be overcome through novel approaches such use of different technology, e.g. mHealth using smart phones. Studies have shown that basic electronic information systems have been used by community health officers and community members to report health indices [33–36]. It is likely that LCS can be trained to provide the required electronic data to NHIS. The other potential challenge is the proposed reimbursement system for NHIS in Ghana that provides an advance payment accredited health service providers for each individual assigned to them over a fixed period of time (capitation payment mechanism). Under this mechanism, it is likely that NHIS subscribers will not choose the LCS as their primary service provider since the services of LCS is limited in scope. However, since malaria is the leading cause of morbidity in Ghana and LCS are the first point of call for febrile illnesses, a pragmatic approach such as exemption of LCS from the capitation mechanism should be carefully tested in order to meet the needs of community members. Conversely, a special package of capitation payment can be piloted in LCS to meet the needs of community members. In addition to such a package, there is the need for a strong referral system to ensure LCS refer patients to higher facilities after they have been treated with ACTs and symptoms still persist. The referral system should be audited by health authorities to avoid the risk of misdiagnosis at the LCS.

### Study limitations

This study has some limitation as it was nested into a study which provided the LCS with mRDT at subsided price and sold it to customers at very low price. This limitation may have influenced the customers’ perception of the “highest and lowest price that they are willing to pay”. The study sample size was small and therefore may not be representative of the entire population of those who seek care from LCS. The study results may be potentially biased and reflective of a largely homogeneous population in a rural area. However, the study setting is typical of other rural areas in Ghana were availability of mRDT at LCS is required most. Another potential source of bias may arise from participants over estimation of their willingness to pay as a result of their intense need or anticipation for quality malaria care. Currently, the use of mRDT by LCS is a policy in Ghana and it will therefore be important to repeat this study in a real-life setting.

## Conclusion

Community members were willing to pay for mRDT and LCS are willing to sell mRDTs. However, a high cost of the mRDT is likely to be a deterrent to community members who are willing to patronize mRDT. The authors suggest to review options for subsidizing the price of mRDT at LCS.

## Abbreviations

mRDT: malaria rapid diagnostic test
KHRC: Kintampo Health Research Center
LCS: licenses chemical shop
LCSs: licenses chemical shops
